# Grain Size Distribution Analysis of Different Activator Doped Gd_2_O_2_S Powder Phosphors for Use in Medical Image Sensors

**DOI:** 10.3390/s22228702

**Published:** 2022-11-11

**Authors:** Panagiotis Liaparinos, Christos Michail, Ioannis Valais, George Fountos, Athanasios Karabotsos, Ioannis Kandarakis

**Affiliations:** 1 Radiation Physics, Materials Technology and Biomedical Imaging Laboratory, Department of Biomedical Engineering, University of West Attica, Ag. Spyridonos, 12210 Athens, Greece; 2 Department of Conservation of Antiquities and Works of Art, University of West Attica, Ag. Spyridonos, 12210 Athens, Greece

**Keywords:** powder phosphors, doping effects, grain size distribution

## Abstract

The structural properties of phosphor materials, such as their grain size distribution (GSD), affect their overall optical emission performance. In the widely used gadolinium oxysulfide (Gd_2_O_2_S) host material, the type of activator is one significant parameter that also changes the GSD of the powder phosphor. For this reason, in this study, different phosphors samples of Gd_2_O_2_S:Tb, Gd_2_O_2_S:Eu, and Gd_2_O_2_S:Pr,Ce,F, were analyzed, their GSDs were experimentally determined using the scanning electron microscopy (SEM) technique, and thereafter, their optical emission profiles were investigated using the LIGHTAWE Monte Carlo simulation package. Two sets of GSDs were examined corresponding to approximately equal mean particle size, such as: (i) 1.232 μm, 1.769 μm and 1.784 μm, and (ii) 2.377 μm, 3.644 μm and 3.677 μm, for Tb, Eu and Pr,Ce,F, respectively. The results showed that light absorption was almost similar, for instance, 25.45% and 8.17% for both cases of Eu dopant utilizing a thin layer (100 μm), however, given a thicker layer (200 μm), the difference was more obvious, 22.82%. On the other hand, a high amount of light loss within the phosphor affects the laterally directed light quanta, which lead to sharper distributions and therefore to higher resolution properties of the samples.

## 1. Introduction

**Figure 1:**
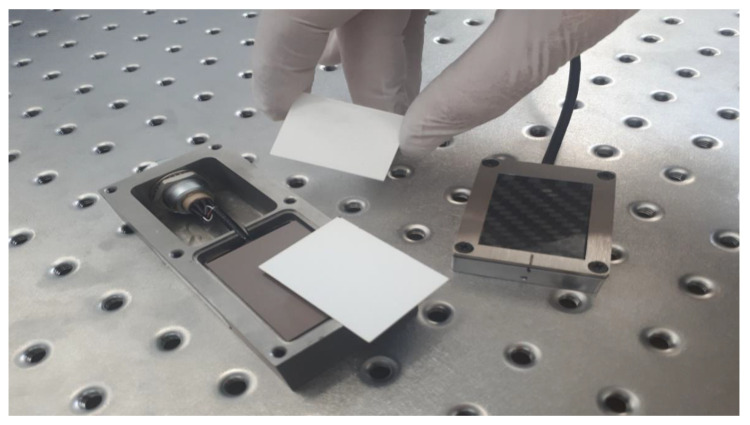
Non-destructive testing CMOS imaging sensors coupled with Gd_2_O_2_S phosphor screens.

In a wide variety of medical imaging systems, optical sensors (i.e., complementary metal-oxide semiconductors [CMOS], charge-coupled devices [CCD], etc.) are employed in combination with phosphor–scintillator layers to form an ionizing radiation sensor or detector [1,2]. In the first case, the detector is consistently found in phosphor-based form where the physical and luminescent properties of the phosphor affect the optical signal transfer and contribute to a high quality of image acquisition. Gadolinium oxysulfide (Gd_2_O_2_S) host material activated with terbium (Gd_2_O_2_S:Tb), europium (Gd_2_O_2_S:Eu), or praseodymium, cerium, and fluorine (Gd_2_O_2_S:Pr,Ce,F), has, until now, been incorporated as the most efficient X-ray-to-light converter in many medical imaging modalities [1,2,3]. For example, (i) Gd_2_O_2_S:Tb powder phosphors are employed in X-ray projection [4] imaging (radiation imaging) and portal imaging systems (radiotherapy), and (ii) Gd_2_O_2_S:Pr ceramic phosphors are applied in X-ray computed tomography systems [5]. Figure 1 shows examples of non-destructive testing (NDT) CMOS imaging sensors, adopted for dual energy medical imaging applications and coupled with Gd_2_O_2_S:Tb phosphor screens [6,7,8].

In recent years, in the field of material science, several preparation methods have been developed and successfully applied for the synthesis of powder phosphors [9]. Most of the time, construction of the phosphor materials and their corresponding properties (e.g., layer thickness, particle sizes and schemes, activator dopant concentrations, absorbing dye incorporation, etc.) have remained trade secrets from the manufacturers [10,11]. On the other hand, a crucial issue in phosphor material research for advanced luminescent properties is the description of the material characteristics in detail in order to implement an accurate methodological approach which provides plausible results. Therefore, an important component in phosphor research and development is the necessary information to feed the methodological approach (e.g., the experimental measurements or the theoretical analysis). In the same vein, for computational optical diffusion modeling, a significant requirement is the entry of input data related to the structural properties of the material, especially when the assumption of a phosphor layer composed of identical particles of a specific size (either corresponding to a mean size taken from previous published works or manufacturer datasheets) is dominant.

Our previous investigations examined the influence of grain size distribution (GSD) on Gd_2_O_2_S light emission properties. In particular, the optical performance was evaluated on the well-known commercial mammographic cassette (Min-R) [12], considering layers consisting of particles (i) of identical grain sizes and (ii) following theoretical GSD configurations (Poisson, Gaussian) [11]. In both studies, the variations of light emission (amount and distribution) showed the importance of considering the exact GSD in light emission modeling where diffusion is described through the light ray interactions with the phosphor grains. In the present work, GSD’s effects on light spreading were assessed for a variety of commercial phosphors purchased from Phosphor Technology (England, UK) [13]. In all cases, Gd_2_O_2_S was the powder phosphor material under investigation; however, phosphor samples (six in total) were synthesized with different dopant activators (i.e., Tb, Eu and Pr,Ce,F dopants) and also presented different GSD for a certain activator. The determination of the role of activator doping effects in grain-size distributed phosphors and their impact on light emission and imaging performance were the main contributions that this paper sought to make. Activator doping effects have been included in many previous studies [14,15], where their influences on the emission spectrum, emission efficiency/light yield, and decay time were examined, without, however, taking into account the grain size distribution. The GSD data of the materials were estimated from scanning electron microscopy (SEM) micrographs. SEM techniques are widely accepted as appropriate and accurate for such purposes, being employed even in more demanding submicron applications [16]. In order to incorporate the GSDs in the optical diffusion model, we considered it more appropriate to simulate the multiple light ray intrinsic interactions with particles within the framework of [17]: (i) Monte Carlo techniques [18] for the simulation of each light ray track separately and (ii) Mie scattering theory to derive the required optical parameters to feed the intrinsic stages of light propagation. This was accomplished with the help of the LIGHTAWE Monte Carlo simulation package [19]. The simulation model was carried out by considering an X-ray beam of 25 keV upon two phosphor layers with thicknesses of 100 μm and 200 μm, respectively. The optical emission performance was evaluated and compared between the samples in terms of (i) the total amount of light emitted and (ii) the light distributed at both exit sides of the phosphor layers, i.e., the reflection and transmission mode.

## 2. Materials and Methods

### 2.1. Synthesis Analysis and Characterization

**Figure 2:**
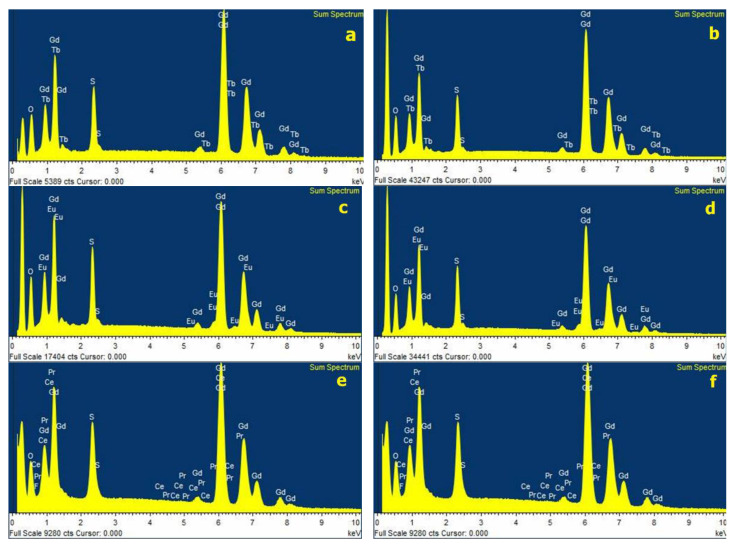
EDX spectra: (**a**) Gd_2_O_2_S:Tb UKL65/UF-R1, (**b**) Gd_2_O_2_S:Tb UKL65/F-R1, (**c**) Gd_2_O_2_S:Eu UKL63/UF-R1, (**d**) Gd_2_O_2_S:Eu UKL63/N-R1, (**e**) Gd_2_O_2_S:Pr,Ce,F UKL59CF/F-R1, and (**f**) Gd_2_O_2_S:Pr,Ce,F UKL59CF/S-R1 phosphors.

Samples of Gd_2_O_2_S:Tb (code numbers: UKL65/UF-R1 and UKL65/F-R1), Gd_2_O_2_S:Eu (code numbers: UKL63/UF-R1 and UKL63/N-R1), and Gd_2_O_2_S:Pr,Ce,F (code numbers: UKL59CF/F-R1 and UKL59CF/S-R1) phosphors were purchased from Phosphor Technology (England, UK) [13]. The size distribution of the grain particles, as well as the energy dispersive X-ray (EDX) elemental spectra of the Gd_2_O_2_S:Tb, Gd_2_O_2_S:Eu, and Gd_2_O_2_S:Pr,Ce,F phosphors were measured with a scanning electron microscope (Jeol JSM 6510LV), as shown in Figure 2. The accelerating voltage was 20.00 kV. For the elemental analysis of particles, the carbon thread evaporation process was employed. Carbon was flash evaporated under vacuum conditions to produce films suited for the specimens in a BAL-TEC CED 030 carbon evaporator (~10^−2^ mbar) [20].

The corresponding stoichiometric results are summarized in Table 1. These results showed the following percent weights for the chemical elements within Gd_2_O_2_S:Tb, Gd_2_O_2_S:Eu, and Gd_2_O_2_S:Pr,Ce,F grains: The dominant element is gadolinium (Gd) ranging from 74.62% to 78.53% weight, then oxygen (O) ranging from 12.09% to 15.47%, sulphur (S) ranging from 6.81% to 8.68%, terbium (Tb) ranging from 1.74% to 2.99%, europium (Eu) ranging from 0.92% to 1.46%, cerium (Ce) 0.39%, praseodymium (Pr) (0.31–0.32%), and fluorine (F) (0.21–0.25%) activators, respectively.

**Figure 3:**
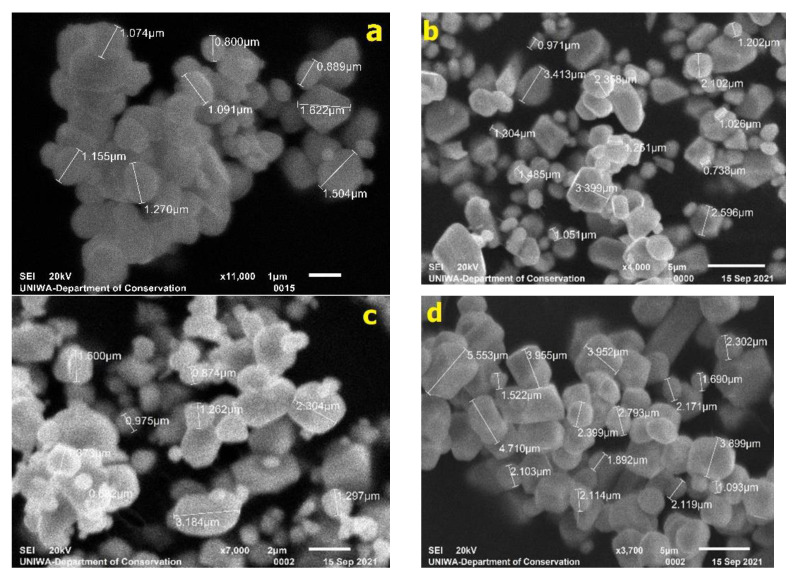

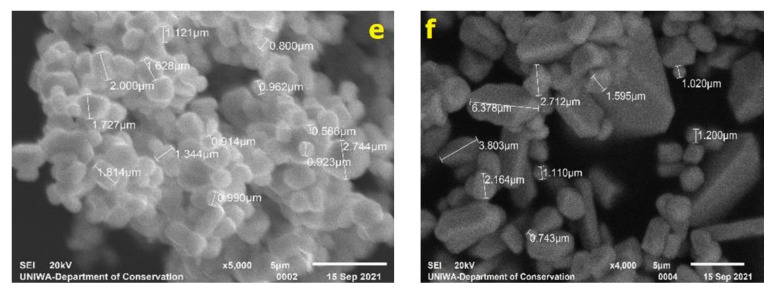
SEM fragments from the: (**a**) Gd_2_O_2_S:Tb UKL65/UF-R1, (**b**) Gd_2_O_2_S:Tb UKL65/F-R1, (**c**) Gd_2_O_2_S:Eu UKL63/UF-R1, (**d**) Gd_2_O_2_S:Eu UKL63/N-R1, (**e**) Gd_2_O_2_S:Pr,Ce,F UKL59CF/F-R1, and (**f**) Gd_2_O_2_S:Pr,Ce,F UKL59CF/S-R1 phosphors.

Figure 3 shows the SEM micrographs obtained from sites of interest within the Gd_2_O_2_S:Tb, Gd_2_O_2_S:Eu, and Gd_2_O_2_S:Pr,Ce,F phosphors samples. All resolvable particles were measured manually in order to avoid any decrease in accuracy due to automatic identification software, in non-spherical and aggregated particles [21,22]. In non-spherical grains, Feret’s diameter method was used to estimate particles’ diameter, measured in an arbitrarily fixed orientation [23,24].

### 2.2. Description of the Monte Carlo Model

**Figure 4:**
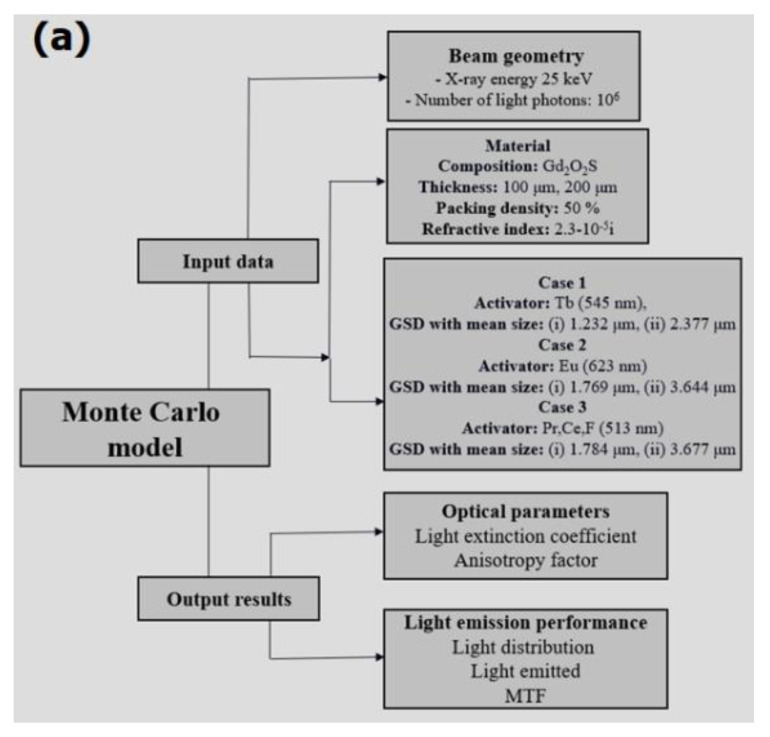

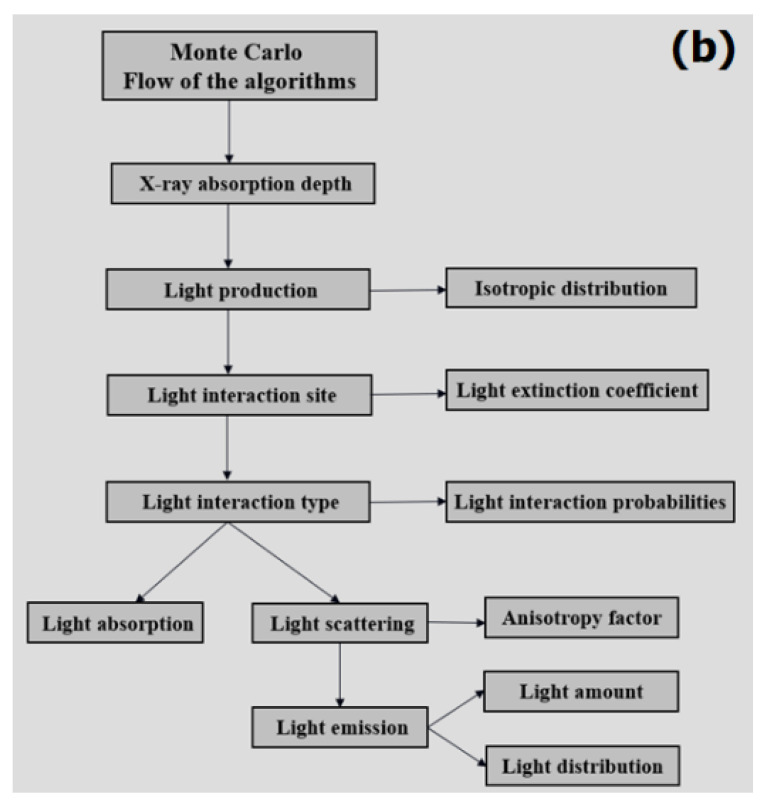
A schematic illustration of the basic structural aspects of the Monte Carlo model: (**a**) input data and output results; (**b**) the flow of the Monte Carlo algorithms.

A schematic representation of the components that make up the model is provided in Figure 4.

The Monte Carlo model has been developed to simulate the light emission properties of the Gd_2_O_2_S luminescent material under the excitation of an X-ray beam of energy 25 keV. Three different cases of activator doping were examined as follows: (i) Tb activator of light wavelength 545 nm, (ii) Eu activator of light wavelength 623 nm, and (iii) Pr,Ce,F activators of light wavelength 513 nm.

Two sets of GSDs were examined corresponding to approximately equal mean particle sizes as provided in Table 2 (Section 3.1), namely (i) 1.232 μm, 1.769 μm, and 1.784 μm; (ii) 2.377 μm, 3.644 μm, and 3.677 μm, for Tb, Eu, and Pr,Ce,F, respectively. The structural and optical properties considered to perform the optical emission are summarized below: (a) packing density 50%, (b) complex refractive index 2.3 × 10^−5^i, and (c) refractive index of the medium 1.35. Two phosphor layers (i.e., layers of 100 μm and 200 μm) were investigated so as to compare the optical diffusion characteristics in thin and thick layers, respectively. A large number of light photons (10^6^) was considered to be created after X-ray-to-light conversion, and thereafter light photons were diffused within the layer until their escape after their interactions with the phosphor particles.

## 3. Results

### 3.1. The Structural Properties of the Phosphor Samples

**Figure 5:**
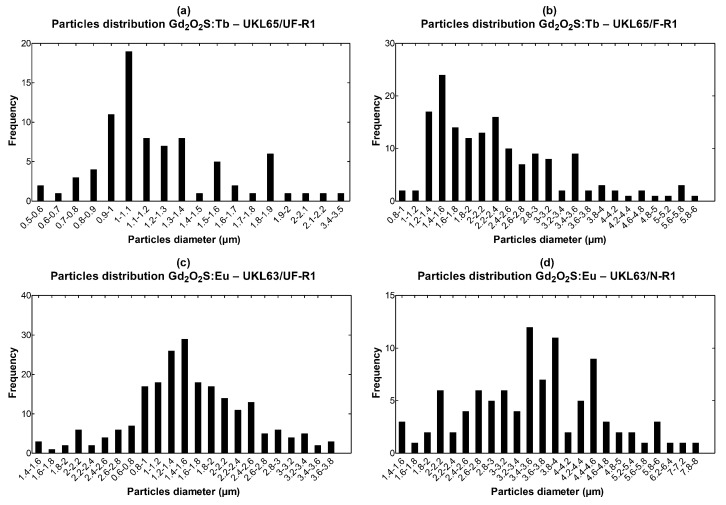

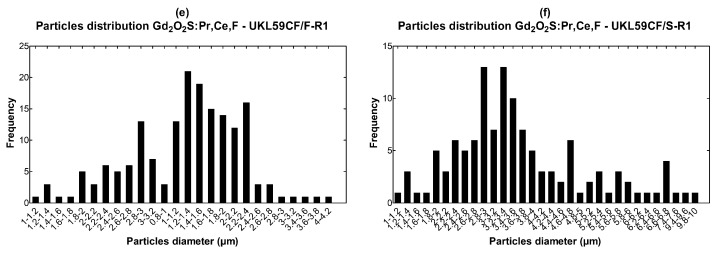
Grain size distributions of the: (**a**) Gd_2_O_2_S:Tb UKL65/UF-R1, (**b**) Gd_2_O_2_S:Tb UKL65/F-R1 (**c**) Gd_2_O_2_S:Eu UKL63/UF-R1, (**d**) Gd_2_O_2_S:Eu UKL63/N-R1, (**e**) Gd_2_O_2_S:Pr,Ce,F UKL59CF/F-R1, and (**f**) Gd_2_O_2_S:Pr,Ce,F UKL59CF/S-R1 phosphor samples.

The estimation of GSDs was based on data obtained from the SEM fragments, as shown in Figure 5. Table 2 shows the corresponding descriptive statistics of the examined grain samples.

The corresponding mean particle sizes were as follows. For Gd_2_O_2_S:Tb (code number: UKL65/UF-R1) it was found equal to 1.232 μm (standard deviation = 0.424); for Gd_2_O_2_S:Tb (code number: UKL65/F-R1) it was found equal to 2.377 μm (standard deviation = 1.031); for Gd_2_O_2_S:Eu (code number: UKL63/UF-R1) it was found equal to 1.769 μm (standard deviation = 0.701); for Gd_2_O_2_S:Eu (code number: UKL63/N-R1) it was found equal to 3.644 μm (standard deviation = 1.170); for Gd_2_O_2_S:Pr,Ce,F (code number: UKL59CF/F-R1) it was found equal to 1.784 μm (standard deviation = 0.566); for Gd_2_O_2_S:Pr,Ce,F (code number: UKL59CF/S-R1) it was found equal to 3.677 μm (standard deviation = 1.542). From Table 1 and Table 2 it can be seen that the average particle size of Gd_2_O_2_S varies with the concentration of the activator and the synthesis method [25]. For Gd_2_O_2_S:Tb, when the concentration of the Tb activator increases from 1.74 to 2.99, the average particle size also increases from 1.232 to 2.377 μm. This is also the case for Gd_2_O_2_S:Pr,Ce,F in which when the total concentration of the Pr,Ce,F activators increases from 0.91 to 0.96, and the average particle size also increases from 1.784 to 3.677 μm. However, in Gd_2_O_2_S:Eu, an opposite behavior is observed, since when the concentration of the Eu activator increases from 0.92 to 1.46, then the average particle size decreases from 3.644 to 1.769 μm.

### 3.2. Evaluation of Required Optical Parameters to Feed the Model

**Figure 6:**
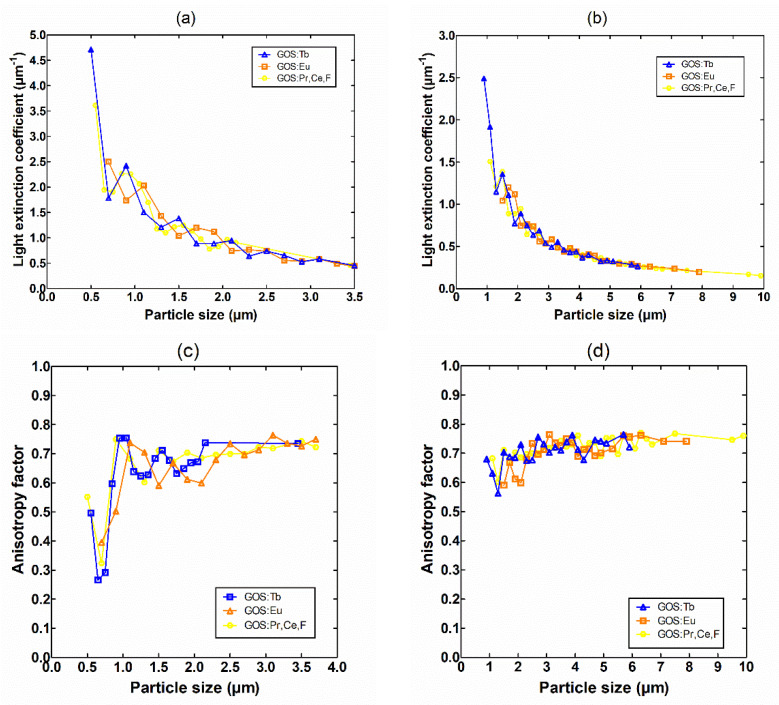
The variation of the (**a**,**b**) light extinction coefficient and (**c**,**d**) anisotropy factor, are illustrated for the GSD distributions provided in Figure 4 of Part 1 of this work. Two comparable sets of GSDs of approximately similar size were considered: (i) 1.232 μm, 1.769 μm, and 1.784 μm (left side), and (ii) 2.377 μm, 3.644 μm, and 3.677 μm (right side), for Tb, Eu, and Pr,Ce,F, respectively.

Figure 6 depicts the variation of the (a,b) light extinction coefficient and (c,d) anisotropy factor as a function of particle size (diameter) of the phosphor samples. The range of particle sizes for each sample is given according to the analysis of their GSDs provided in Figure 4. The evaluation of the optical parameters was based on Mie scattering theory. The mathematical expressions used to predict the optical parameters are given in Appendix A.

### 3.3. Light Emission Performance of Phosphor Samples

**Figure 7:**
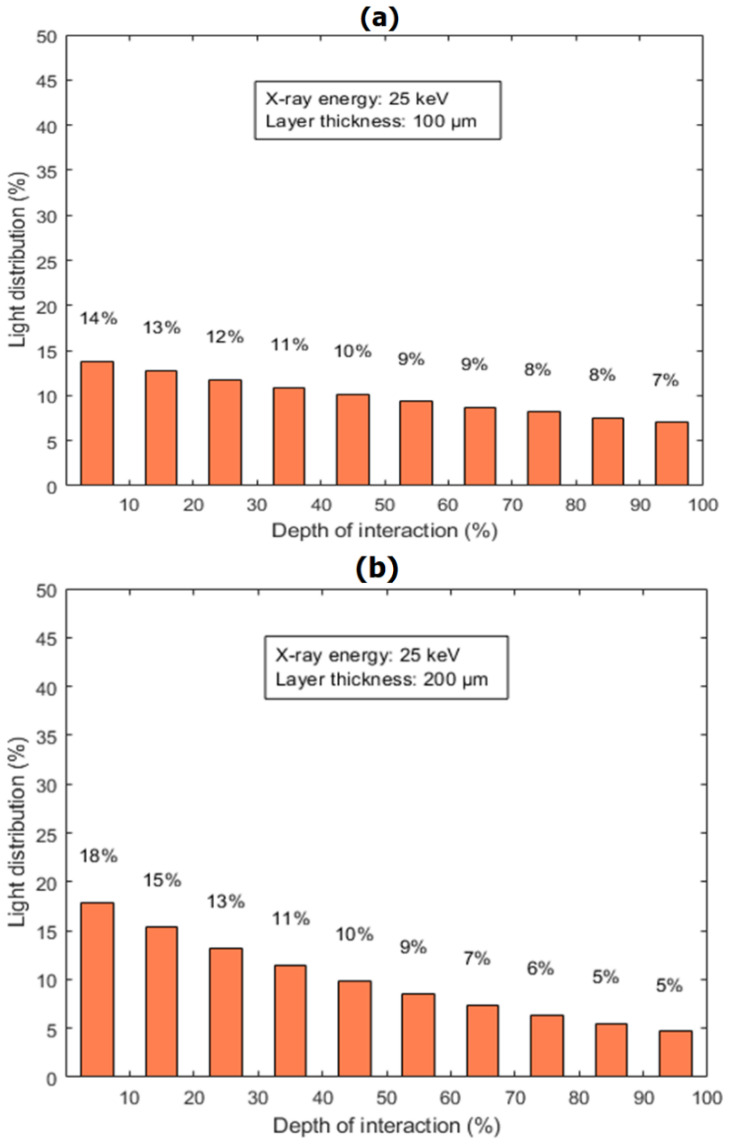
The light distribution (%) as a function of the depth of the interaction site (the percentage interaction depth with respect to the total screen thickness) within the phosphor layer of Gd_2_O_2_S phosphor material irradiated by an X-ray beam of 25 keV upon a phosphor layer of thickness: (**a**) 100 μm (upper image) and (**b**) 200 μm (lower image).

**Figure 8:**
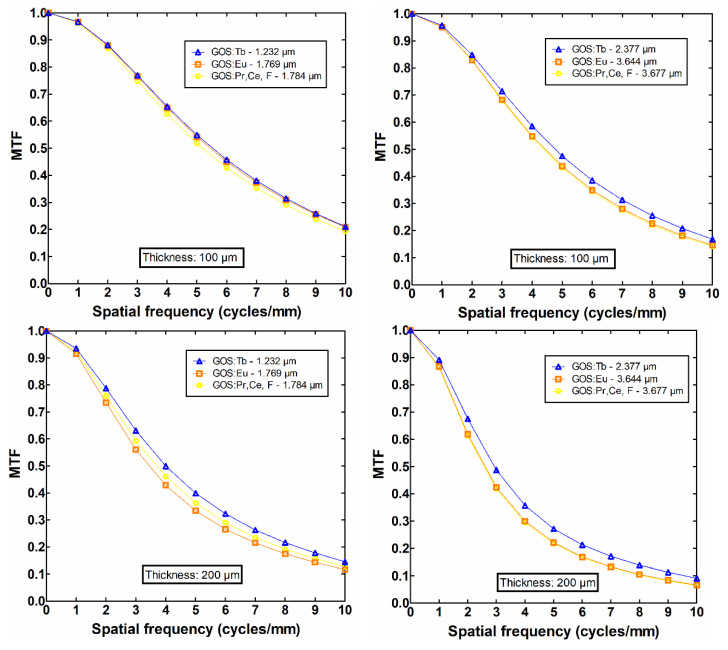
MTF curves of two phosphor layers, 100 μm (**upper images**) and 200 μm (**lower images**). Two comparable sets of GSDs of approximately similar size were considered: (i) 1.232 μm, 1.769 μm, and 1.784 μm (**left side**), and (ii) 2.377 μm, 3.644 μm, and 3.677 μm (**right side**), for Tb, Eu, and Pr,Ce,F, respectively.

The light emission performance was assesed considering two cases of light production. The distributed sites of light production were obtained by assuming an X-ray beam of 25 keV upon two phosphor layers: (i) a thin layer of 100 μm and (ii) a thick layer of 200 μm. These values are typically used in mammography and radiography applications, respectively. The distribution of light production as a function of depth within the phosphor layer is provided in Figure 7. In both cases, the sites of light production are determined by the sites of X-ray absorption, which follow the exponential law of radiation (X-ray) attenuation within the layer. The mass attenuation coefficient was taken as equal to 20.32 cm^2^/g (a value which corresponds to the X-ray attenuation properties of Gd_2_O_2_S at 25 keV assuming a density of 7.34 g/cm^3^). In order to compare and estimate the light diffusion characteristics between the different GSDs configurations, both the spatial distribution and the amount of light were evaluated in terms of (a) the modulation transfer function (MTF) curves shown in Figure 8 and (b) the percentage of light emitted relative to light produced (reflection and transmission mode) as given in Table 3 and Table 4.

The difference in light distribution, expressed through MTF and resolution, is mainly due to the light interaction mechanisms within the powder layer. The MTF curves were obtained from the full width at half maximum (FWHM) of the emitted light point spread function (PSF) and are related to the angular distribution of the light diffusion towards the output surface of the layer. The MTF curves and the amount of light emitted were evaluated assuming two comparable sets of GSDs of approximately similar size: (i) 1.232 μm, 1.769 μm, and 1.784 μm and (ii) 2.377 μm, 3.644 μm, and 3.677 μm, corresponding to the Tb, Eu, and Pr,Ce,F activators, respectively. Based on the MTF curves, at 10% MTF, the following specific numerical estimations of resolution were found: (i) 12.9 cycles/mm for 1.232 μm, which was similar to 1.769 μm and 2.4% higher than that of 1.784 μm, considering the thin layer of 100 μm. However, for the thick layer of 200 μm, the resolution was found to be 11.6 cycles/mm for 1.232 μm, which was 8.7% higher than that of 1.769 μm and 4.5% higher than that of 1.784 μm; (ii) 12.1 cycles/mm for 2.377 μm, which was 4.5% higher than that of 3.644 μm and 5.6% higher than that of 3.677 μm, considering the thin layer of 100 μm. However, for the thick layer of 200 μm, the resolution was found 9.6 cycles/mm for 2.377 μm, which was 16.3% higher than that of 3.644 μm and 17.5% higher than that of 3.677 μm. In both sets of GSDs, the difference in light distribution seemed to be low when investigating the thin phosphor layers; nevertheless, the difference was found to increase in thick layers due to the larger number of total light photon interactions with the phosphor particles.

## 4. Discussion

The evaluation of light propagation and emission is directly dependent on (i) the sites of light production within the layer and (ii) the total number of light ray interactions (scattering and absorption) with the phosphor particles. Both factors alter the amount and direction of light tranjectories and affect the configuration of light transport within the material until the arrival of light photons at the output surface of the layer. On the one hand, the sites affect the distance for light photons to reach the exit surfaces, while on the other hand, the number of interactions influence the amount of light absorption which in turn greatly affects the laterally directed light quanta (due to their longer trajectories) and consequently the spatial distribution of emitted light photons. The aforementioned procedures are the main processes that cause significant changes to the optical difussion properties of phosphor materials, and their suitability for use in medical imaging sensors depends on the way they are treated.

Regarding the first process, the distributed sites of light production depend on the thickness of the phosphor layer. Based on the results illustrated in Figure 7, the fraction of produced optical photons was approximately evaluated as (a) 14% at a layer thickness of 100 μm compared to 18% at a layer thickness of 200 μm considering the first sublayer and (b) 7% at a layer thicnkess of 100 μm compared to 5% at a layer thicnkess of 200 μm considering the last sublayer. These results showed that in thick layers a higher fraction of light is produced at sites away from the back side of the layer (i.e., the non-irradiatate output surface). As a result, the amount of light emitted in transmission mode is considerably lower in thick layers (200 μm) due to the long light photon trajectories until escape. This can be also verified by numerical data provided in Table 3 and Table 4 for all cases. For instance, the amount of light emitted was estimated to be higher at a layer thickness of 100 μm compared to 200 μm. An increase of approximately (a) 111.8% and 62.5% considering the mean size of 1.232 μm and 2.377 μm, respectively, was found for Tb dopant Gd_2_O_2_S; (b) 68.0% and 45.1% considering a mean size of 1.769 μm and 3.644 μm, respectively, for Eu dopant Gd_2_O_2_S; and (c) 103.9% and 42.8% considering a mean size of 1.784 μm and 3.677 μm, respectively, for Pr,Ce,F dopant Gd_2_O_2_S. The amount of light emitted is also related to the amount of light absorbed within the phosphor layer, which was observed to be dominant in thicker phosphor layers.

As mentioned above, the increase of light absorption is also due to the second process of multi-intercations of the light beam with the optical scatterers (i.e., the phosphor grains). This is another aspect which plays a critical role in the fluctuations of the light emitted (either in the amount or distribution). According to Table 3 and Table 4, light absorption was found (i) to be almost similar, 24.51% and 25.45%, for Tb and Eu dopants considering the first set of GSDs (mean size of 1.232 μm and 1.769 μm) and the thin layer (100 μm), while for the thicker layer (200 μm) the difference was more obvious (50.52% and 42.15%, respectively); (ii) to be almost similar, 8.17% and 8.46%, for Eu and Pr,Ce,F dopants considering the second set of GSDs (mean size of 3.644 μm and 3.677 μm) and the thin layer (100 μm), while for the thicker layer (200 μm), light absorption was slightly different (22.82% and 21.89%, respectively). Apart of the amount of light loss within the phosphor, the higher number of light interactions (i.e., light suffers higher attenuation) affects the laterally directed light quanta, which leads to sharper distributions and therefore to higher resolution properties, as shown in Figure 8 and analyzed in Section 3.3. The intrinsic mechanisms of light attenuation are based on the optical parameters, which are given in Figure 6. In small-sized GSDs, the light extinction coefficient takes high values contributing to the increase of light interactions (light absoprtion is higher for the first set of samples where the mean particle size is low). In addition, the oscillations observed in the optical extinction are due to the dependence of the extinction efficiency factor *Q_ext_* on particle size (Equation A1 in Appendix A). The type of dopant for phosphor synthesis can alter the GSD of the phosphor material, as shown in Figure 5. GSDs with small particles will provide lower intensity in the scintillation emission due to scattering, and on the other hand, screens with large particles tend to lose homogeneity [26]. Advanced structural properties could be achieved, owing to particle shape and size based on the synthesis methods, e.g., vapor transport (evaporation/condensation), surface diffusion, lattice (volume) diffusion, grain boundary diffusion, and dislocation motion [27], as well as their corresponding preparation intrinsic mechanisms and processing conditions (temperature, particle size, applied pressure, particle packing, etc.). The presence of impurities also changes the luminescence properties of the compounds [28]. Furthermore, particle shape affects the packing of the so-called “green body” of the phosphor structure, and possible deviations from the spherical shape lead to increased porosity along with reduced packing density [29]. The finding that the type of activator provides different GSD could be traced to the aforementioned synthesis methods and processing conditions, however, up to now, there is no clear understanding of the exact relation of the particular GSD shape with the dopant usage. A future research direction could be the analysis of a high number of GSDs of phosphor samples (i.e., repeatable preparation of future tests is needed) so as to find a potential dependence between the type of the dopant and the GSD configuration. The results of the present analysis could be useful in the design of new sensors with improved performance, both in terms of sensitivity (i.e., emission efficiency) and medical image quality.

## 5. Conclusions

The GSD of powder phosphors is affected by several parameters according to the synthesis methods and processing conditions used during the development of the phosphor layers. One of these parameters is the type of dopant, which plays a significant role in the particle shape and size configuration. This role and its impact on the light emission and imaging performance were the main contributions that this paper sought to achieve.

In the present study, we tried to investigate a series of Gd_2_O_2_S powder phosphors doped with different types of dopants (e.g., Tb, Eu, and Pr,Ce,F). The analysis of phosphor samples showed different GSDs based on the type of activator. Our Monte Carlo optical difussion model showed almost similar light absorption in some cases, e.g., 24.51% and 25.45% for Tb and Eu dopants and 8.17% and 8.46% for Eu and Pr,Ce,F dopants considering the thin layer (100 μm). However, for the same conditions, the difference was more obvious, 50.52% and 42.15%, and slightly different, 22.82% and 21.89%, when examining the thicker layer (200 μm). It was also found that there was a direct dependence of light loss upon the distribution of light emission, which, therefore, affected the resolution properties of the phosphor layers. Our results indicate that in powder phosphor investigations, apart from the optical properties of the dopant (concentration and wavelength emission), one should also take into account the effect on the structural properties due to modifications of the particle sizes within the phosphor layer.

## Figures and Tables

**Table 1:** Stoichiometric results for the examined grains.

Chemical Element	Gd_2_O_2_S:Tb UKL65/UF-R1	Gd_2_O_2_S:Tb UKL65/F-R1	Gd_2_O_2_S:Eu UKL63/UF-R1	Gd_2_O_2_S:Eu UKL63/N-R1	Gd_2_O_2_S:Pr.Ce.F UKL59CF/F-R1	Gd_2_O_2_S:Pr.Ce.F UKL59CF/S-R1
Gadolinium (Gd)	78.53	78.11	74.62	77.07	76.63	75.48
Oxygen (O)	12.82	12.09	15.24	13.39	14.47	15.47
Sulphur (S)	6.90	6.81	8.68	8.62	7.99	8.09
Terbium (Tb)	1.74	2.99	-	-	-	-
Europium (Eu)	-	-	1.46	0.92	-	-
Praseodymium (Pr)	-	-	-	-	0.31	0.32
Cerium (Ce)	-	-	-	-	0.39	0.39
Fluorine (F)	-	-	-	-	0.21	0.25
Totals	100	100	100	100	100	100

**Table 2:** Descriptive statistics for Gadolinium Oxysulphide phosphor grains.

Sample Descriptive Statistics	Gd_2_O_2_S:Tb UKL65/UF-R1	Gd_2_O_2_S:Tb UKL65/F-R1	Gd_2_O_2_S:Eu UKL63/UF-R1	Gd_2_O_2_S:Eu UKL63/N-R1	Gd_2_O_2_S:Pr,Ce,F UKL59CF/F-R1	Gd_2_O_2_S:Pr,Ce,F UKL59CF/S-R1
Mean grain size (μm)	1.232	2.377	1.769	3.644	1.784	3.677
Standard error	0.046	0.081	0.050	0.117	0.051	0.139
Median grain size (μm)	1.105	2.170	1.604	3.582	1.675	3.338
Mode grain size (μm)	N/A	1.473	1.435	N/A	1.766	3.326
Standard deviation	0.424	1.031	0.701	1.170	0.566	1.542
Sample variance	0.180	1.064	0.491	1.368	0.321	2.377
Kurtosis	8.809	1.589	0.038	1.621	2.082	2.885
Skewness	2.163	1.286	0.760	0.804	1.130	1.362
Range (μm)	2.925	5.000	3.118	6.552	3.183	8.817
Minimum grain size (μm)	0.566	0.894	0.609	1.422	0.884	1.162
Maximum grain size (μm)	3.492	5.894	3.727	7.974	4.067	9.979
Counted grains	83	162	196	100	125	123

**Table 3:** Light emission performance (absorption, transmission, and reflection) of different GSD configurations: (i) Gd_2_O_2_S:Tb (1.232 μm), (ii) Gd_2_O_2_S:Eu (1.769 μm), (iii) Gd_2_O_2_S:Pr,Ce,F (1.784 μm), (iv) Gd_2_O_2_S:Tb (2.377 μm), (v) Gd_2_O_2_S:Eu (3.644 μm), and (vi) Gd_2_O_2_S:Pr,Ce,F (3.677 μm). Light performance is given by the light quanta produced from an X-ray beam with an energy of 25 keV upon a phosphor layer with a thickness of 100 μm.

	Light Emission Performance		
	Absorption (%)	Reflection (%)	Transmission (%)
		Thickness: 100 μm	
Gd_2_O_2_S:Tb (1.232 μm)	24.51	43.3	32.19
Gd_2_O_2_S:Eu (1.769 μm)	25.45	42.74	31.81
Gd_2_O_2_S:Pr,Ce,F (1.784 μm)	19.23	45.94	34.83
Gd_2_O_2_S:Tb (2.377 μm)	12.44	49.33	38.23
Gd_2_O_2_S:Eu (3.644 μm)	8.17	51.37	40.46
Gd_2_O_2_S:Pr,Ce,F (3.677 μm)	8.46	51.1	40.44

**Table 4:** Light emission performance (absorption, transmission, and reflection) of different GSD configurations: (i) Gd_2_O_2_S:Tb (1.232 μm), (ii) Gd_2_O_2_S:Eu (1.769 μm), (iii) Gd_2_O_2_S:Pr,Ce,F (1.784 μm), (iv) Gd_2_O_2_S:Tb (2.377 μm), (v) Gd_2_O_2_S:Eu (3.644 μm), and (vi) Gd_2_O_2_S:Pr,Ce,F (3.677 μm). Light performance is given by the light quanta produced from an X-ray beam with an energy of 25 keV upon a phosphor layer with a thickness of 200 μm.

	Light Emission Performance		
	Absorption (%)	Reflection (%)	Transmission (%)
		Thickness: 200 μm	
Gd_2_O_2_S:Tb (1.232 μm)	50.52	34.28	15.20
Gd_2_O_2_S:Eu (1.769 μm)	42.15	38.92	18.93
Gd_2_O_2_S:Tb (2.377 μm)	46.35	36.57	17.08
Gd_2_O_2_S:Eu (3.644 μm)	31.98	44.49	23.53
Gd_2_O_2_S:Pr,Ce,F (3.677 μm)	22.82	49.19	27.89
Gd_2_O_2_S:Tb (2.377 μm)	21.89	49.69	28.32

## Data Availability

Data is contained within the article.

## Appendix A

Optical diffusion is described through the light ray interactions with the phosphor particles. Within the framework of the Mie scattering theory, the simulation algorithms describe light trajectory in terms of two optical parameters. The first is the light extinction coefficient, *m_ext_*, which denotes the path length that light travels within the materials until interaction with the phosphor particles. The light extinction coefficient is evaluated as follows [13]:

*m_ext_ = V_d_AQ_ext_* (A1)

where *V_d_* is the volume density of the phosphor material, *A* is the geometrical cross-section of the grain, and *Q_ext_* is the extinction efficiency factors. The second optical parameter is the anisotropy scattering factor, *g*_,_, which denotes the direction of the light after scattering with the phosphor particles. The anisotropy factor is obtained according to the Henyey-Greenstein distribution as given below:

(A2) $$g=\frac{\int_{0}^{\pi }2\pi S_{11}\left(\theta \right)cos\theta sin\theta d\theta }{\int_{0}^{\pi }2\pi S_{11}\left(\theta \right)sin\theta d\theta }\;$$

where *S*_11_(*θ*) is the first element of the so-called Mueller matrix, which implies that light extinction is independent of the light polarization state. *S*_11_(*θ*) is related to the complex elements of scattering matrix *S*_1_(*θ*) and *S*_2_(*θ*).
